# 3D chick embryo chorioallantoic membrane model as an *in vivo* model to study morphological and histopathological features of feline fibrosarcomas

**DOI:** 10.1186/s12917-017-1114-4

**Published:** 2017-06-26

**Authors:** Katarzyna Zabielska-Koczywąs, Agata Wojtkowska, Izabella Dolka, Anna Małek, Magdalena Walewska, Anna Wojtalewicz, Artur Żbikowski, Roman Lechowski

**Affiliations:** 1 Department of Small Animal Diseases with Clinic, Faculty of Veterinary Medicine, Warsaw University of Life Sciences, Nowoursynowska 159c, Warsaw, Poland; 2 Department of Pathology and Veterinary Diagnostics, Faculty of Veterinary Medicine, Warsaw University of Life Sciences, Nowoursynowska 159, Warsaw, Poland

**Keywords:** Feline fibrosarcoma, Experimental animal model; *in ovo* assay, Ki-67, PCNA, Immunohistochemistry

## Abstract

**Background:**

The chick embryo chorioallantoic membrane (CAM) model is well described in human medicine as a cost-effective, easy to perform preclinical oncological model for observing pro- and antiangiogenic response, tumor biology and metastasis. The main objective of this article was to present the modification of the CAM assay in order to evaluate tumor growth from two feline fibrosarcoma cell lines (FFS1, FFS3) and describe their morphological and histopathological features.

**Results:**

The authors described morphological and histopathological features of two feline fibrosarcoma cell lines (FFS1 and FFS3) grown on the CAM. Tumors from the FFS1 cell line showed high malignancy (grade III), while tumors from the FFS3 cell line were grade II. Proliferation markers (Ki-67 and PCNA) were determined and the positive correlation between PCNA and tumor grade (*r* = 0.8247; *p* < 0.001) was demonstrated, as opposed to Ki-67.

**Conclusions:**

The results obtained indicate that PCNA may be helpful to evaluate the tumor grade, better than Ki-67, for feline fibrosarcomas. However, further investigations of proliferation marker, in bigger number of feline spontaneous fibrosarcomas and feline fibrosarcomas grown on the CAM from different cell lines, are needed to confirm these observations.

## Background

Feline injection-site sarcomas (FISS) are malignant subcutaneous tumors. The etiopathogenesis is unclear, however, inflammatory responses within the injections site (especially vaccines against rabies and feline leukemia virus - FeLV) may play a role in malignant transformation of the connective tissues cells resulting in sarcoma. FISS usually occurs in middle age cats. The diagnosis is based on history, clinical examination and histopathology [1]. Based on the predominant histogenesis within different FISSs, they can be diagnosed as 8 subtypes: fibrosarcoma, malignant fibrous histiocytoma, osteosarcoma, chondrosarcoma, rhabdomyosarcoma, myxosarcoma, myofibrosarcoma and undifferentiated sarcomas. The most common type is fibrosarcoma (more than 80% of FISSs) [2]. Methods of treatment include: surgery, radiotherapy and/or chemotherapy [3]. As standard chemotherapeutic agents (e.g. doxorubicin, cyclophosphamide) have many adverse side effects and their effectiveness in treatment of FISS is debatable, new substances such as tyrosine kinase inhibitors (masitinib, toceranib), nanoparticles conjunct with cytostatic drugs (Au-GSH-Dox) are under investigation [4–6]. Recently, immunotherapy with Oncept Il-2 has been approved to be used as an adjunctive therapy in addition to surgery and brachytherapy and/or chemotherapy in cats with the first stage of the disease (FISS without enlargement of lymph nodes and metastasis) [7].

In order to assess the effectiveness of new drugs, various in vitro and in vivo preclinical studies are needed. Researchers are looking for new preclinical models as standard rodent models are expensive, time consuming and require approval from the Animal Ethics Commission. The chick embryo chorioallantoic membrane (CAM) model is well-known in human medicine, first described by Rous and Murphy [8]. It is believed to be a cost-efficient, easy to perform model for observing both pro- and anti-angiogenic response [9, 10] and the effectiveness of anticancer agents [11]. In human medicine CAM assay was utilized in studies for colon cancer (SW 680, SW 420) [12], fibrosarcoma (Ht 1080) [13, 14], glioma (U-87 MG) [15], osteosarcoma (MMNG-HOS; SAOS; U2OS) [16], neuroblastoma (IMR 32) [10], nasopharyngeal carcinoma (HONE1; 5-8F; 6-10B; C66-1) [17], ovarian cancer (OVCAR-3, SKOV-3, OV-90) [18] and head and neck squamous cell carcinoma (UM-SCC-29) [19]. However, not every human cell line has the ability to form solid tumors on the CAM. Balke et al. showed that only three out of eight osteosarcoma cell lines formed solid tumors [16]. To our knowledge, there are only a few reports on the use of the CAM model in veterinary medicine [20–24]. The main objective of this article was to present the modification of the CAM assay in order to evaluate tumor growth from two feline fibrosarcoma cell lines (FFS1, FFS3) and describe their morphological and histopathological features. The immunoreactivity of proliferation markers: the Ki-67 antigen and proliferating nuclear cell antigen (PCNA) was assessed, as it has a good prognostic value for chemotherapy response in various tumors (e.g. human and canine mammary tumors, human soft tissue sarcoma, feline lymphoma) [25–27]. In human soft tissue sarcomas and breast cancer Ki-67 proliferating index was positively correlated with histological grade, tumor stage, aggressive behavior and prognosis [28–33]. In veterinary medicine such correlation was also demonstrated in several types of tumors, e.g. canine soft tissue sarcomas and canine mammary gland tumors [28, 29, 34–37]. PCNA is a nuclear protein involved in DNA synthesis and its concentration directly correlates with proliferation in normal or neoplastic tissue. In some tumors the PCNA score correlates with the histological grade and with the immunoreactivity of Ki-67 [34, 38]. There are only a few reports about the Ki-67 expression in canine and feline fibrosarcomas [39, 40].

## Methods

### Cell culture

Feline fibrosarcoma cell lines (FFS1, FFS3) derived in Justus Liebig Universitat in Giessen (Germany) [41] were cultivated under standard aseptic conditions (5% CO_2_, 95% humidity and 37 °C) in Dulbeco’s Modified Eagle Medium (DMEM) with glucose (4500 mg/L) (Gibco BRL), enriched with 10% *v*/v heat inactivated fetal bovine serum (FBS), penicillin-streptomycin (50 mL IU-1), amphotericin B (2.5 mg/mL-1) (reagents obtained from Sigma Aldrich, USA). The medium was changed every 48–72 h, when cell confluence reached 75–80%.

### Growth kinetics

5 × 10^4^ cells of FFS1 and FFS3 cells were seeded per well of the 6-well plate and cultivated with DMEM to evaluate the kinetics of the two cell lines. On days: 2,4,7 and 9 cells were trypsinized and the number of living cells was assessed using Countess II FL Automatic Cell Counter.

### CAM assay

**Fig. 1:**
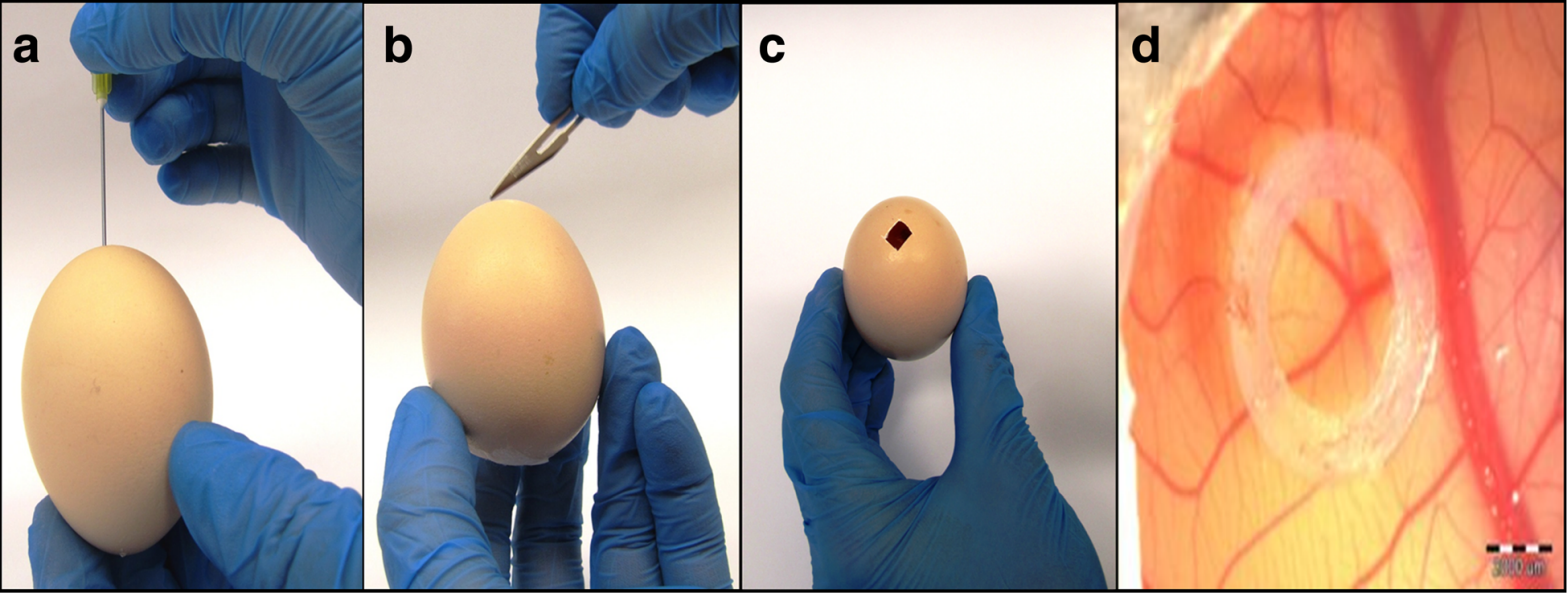
Steps of establishing the CAM model. **a** Day 3 - piercing the eggshell in the blunt end of the egg. **b, c** Day 6 - creating a “window” in the eggshell. **d** Day 6 - placing the silicone ring on the CAM

Ross 308 fertilized chicken eggs (Poultry Hatchery Pankowski Jan, Białobrzegi, Poland) were used to perform the study. The CAM assay was performed according to the procedure described previously [20, 21] with some modifications. On the 3rd day of incubation, the hole in the blunt end of each egg was made (Fig. 1a) and closed with a semitransparent patch and the eggs were turned 180°. On the 6th day of the chick embryos incubation a ‘window’ in the eggshell (7 × 7 mm size) was cut out (Fig. 1b, c). After that sterile silicone rings (7 mm in external diameter, 6 mm in internal diameter, 1 mm thick) (Zegir PTHU, Poland) were put on the chorioallantoic membrane (Fig. 1d). Feline fibrosarcoma cells (5 × 10^6^ cells per egg suspended in 25ul of medium) were aseptically injected into the silicone rings. The amount of cells seeded into ring was determined by performing several studies with various amounts of cells seeded per egg (1 × 10^5^, 5 × 10^5^, 1 × 10^6^, 3 × 10^6^, 5 × 10^6^). In agreement with the studies on the cultivation of human cell lines on the CAM, 5 × 10^6^ was found the most effective amount of cells seeded per egg, as tumor formation was found in 80% of inoculated eggs. Saline and medium alone (25 ul per egg) were used as negative controls. After cell inoculation the ‘windows’ were taped with semitransparent patch of high air and humidity permeability. 24 and 48 h later the eggs were illuminated with an ovoscope to assess the chick embryos vitality. After 10 days (on the 16th day of incubation) tumor growth was observed and then continuously monitored until the end of the experiment (19th day of incubation) using veterinary Digital Macro View™ Otoscope (Welch Allyn, USA). Photographs were taken with a 3D microscope VHX-5000 (Keyence, Belgium) and size of tumors was measured in real time.

### Histopathology

**Fig. 2:**
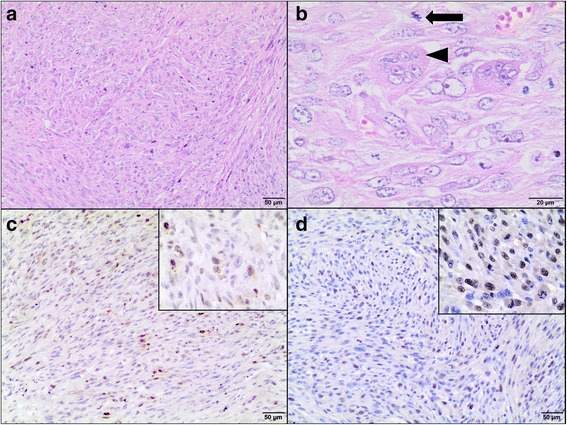
Hematoxillin and eosin staining and immunohistochemistry of spontaneous feline fibrosarcoma. **a** A highly cellular neoplasm composed of spindle shaped to polygonal cells arranged in interlacing streams and bundles and supported by small to moderate amounts of collagenous matrix. H&E, magnification 100×. **b** The neoplastic cells have indistinct cell borders, small amount of fibrillar, pale eosinophilic cytoplasm, some showing slightly vacuolated cytoplasm. They have round to oval nuclei varied in size, dispersed and peripheralized chromatin with prominent magenta nucleoli (1-2). Mitotic figures are up to 8 per high power field (HPF), including bizarre mitotic figures (*arrow*). There are bi- or trinucleated cells as well as multinucleated giant cells (*arrowhead*) containing up to 9-30 and variable amounts of eosinophilic cytoplasm. Necrotic neoplastic cells undergo karyolysis and karyopycnosis are interspersed throughout the tumor. H&E, magnification 400×. **c** Ki-67*-*positive cells (*brown nuclei*) in feline fibrosarcoma. IHC, magnification 100× (400× insert)*.* **d** PCNA*-*positive cells (*brown nuclei*) in feline fibrosarcoma. IHC, magnification 100× (400× insert)

The tumor growth was confirmed by hematoxylin and eosin (H&E) staining. Tumors were graded according to a scale proposed by Couto et al. based on mitotic index (MI), cellular differentiation and the presence and extent of necrosis within the tumor [40]. Tumors were scored from 1 to 3 for overall differentiation, where 1 meant that the neoplasm is well-differentiated, 2 that the tumor was less differentiated but still had a defined histological phenotype and 3 that was poorly differentiated. Mitotic rate per 10 high-power fields (400× magnification) (1 – 1-9 mitosis; 2 – 10-19 mitosis; 3 – >19 mitosis) and intra-tumoral necrosis (1– no necrosis; 2 – necrosis <50% of the total area of the specimen; 3 – necrosis >50% of the total area of the specimen). Final cumulative scores (summary of the individual scores for overall differentiation; mitotic rate and necrosis) were evaluated for each tumor. Cumulative scores of 3 or 4 defined grade I; 5 or 6 – grade II; 7 or more – grade III. Moreover, spontaneous feline fibrosarcoma was used as a positive control (Fig. 2) to confirm that the model described herein recapitulates the hallmarks of spontaneous feline fibrosarcomas.

### Immunohistochemistry (IHC)

**Fig. 3:**
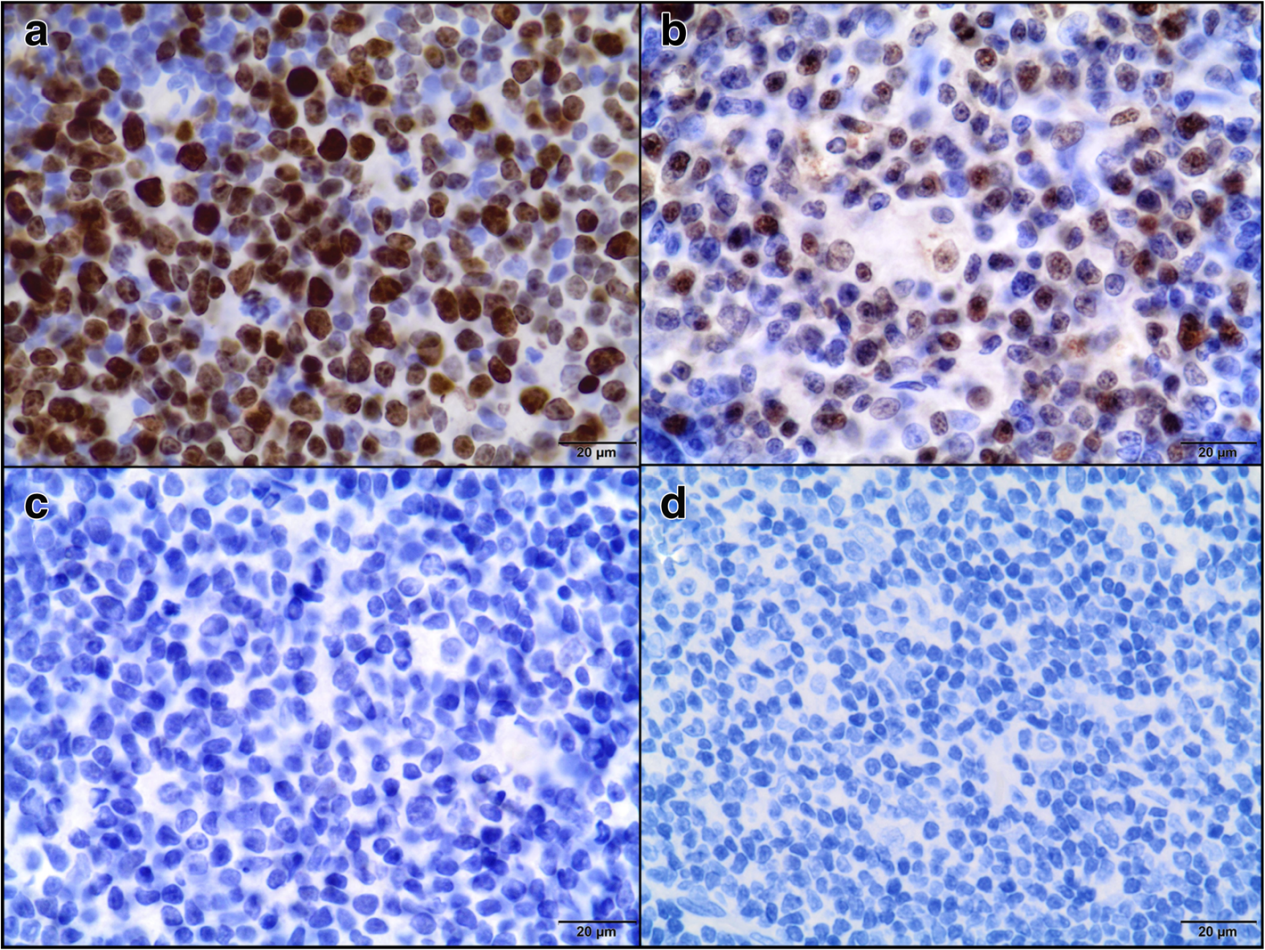
Feline lymph node. Positive control of immunohistochemical reaction for Ki-67 (**a**) and PCNA (**b**) as nuclear pattern (*brown precipitate*). Negative controls for Ki-67 (**c**) and PCNA (**d**). IHC, magnification 400 ×

The tissue samples were fixed in 10% formalin*,* embedded in paraffin blocks, then were cut into 3 μm sections. To minimize the problems arising during performing IHC staining, which may negatively influence the results, obtained time of the tissue fixation was reduced (the tissues were fixed in formalin for approximately 24–48 h and then directly performed for IHC) and fresh reagents were used. Sections were mounted on hydrophilic slides (Hydrophilic Plus Microscope Slides) and baked at 37 °C overnight. After dewaxing in xylene and rehydratation in ethanol, the slides were heated in a microwave in 0.02 M citrate buffer, pH 6.0 for antigen retrieval. After cooling, the sections were incubated in a 3% perhydrol solution for 15 min to block the endogenous peroxidase reaction. Non-specific binding was blocked by incubation in 5% bovine serum albumin (Sigma Aldrich, Germany). After 30 min, the following primary antibodies (diluted in 1% bovine serum) were used: monoclonal mouse anti-proliferating cell nuclear antigen, clone PC10 (Dako, Denmark) diluted 1:100 and monoclonal mouse anti-Ki-67 antigen, clone MIB 1 (Dako, Denmark) diluted 1:75. The slides were incubated in a humidified chamber for 1 h at room temperature. Secondary antibodies were used according to the manufacturer’s instructions. The sections were washed, covered with diaminobenzidine chromogen (DAKO, Denmark) and counterstained with Ehrlich’s hematoxylin for 10 min. They were dehydrated in a graded series of alcohols, cleared in xylene and mounted using the DPX medium (Gurr®, Sigma-Aldrich) and coverslips. Paraffin-embedded tissues of feline lymph nodes were used as positive controls for both Ki-67 (Fig. 3a) and PCNA (Fig. 3b) [42]. Moreover, to validate the CAM model for feline fibrosarcomas, Ki-67 labeling index (LI) and PCNA LI were also assessed in spontaneous feline fibrosarcomas (*n* = 23) obtained from the archives of the Division of Animal Pathomorphology at the Department of Pathology and Veterinary Diagnostics, Faculty of Veterinary Medicine, Warsaw University of Life Sciences.

The staining without the use of primary antibodies was done as a negative control for each immunohistochemical analysis (Fig. 3c, d). The pictures were taken using Olympus microscope BX60 (Olympus, Japan).

### Scoring of immunohistochemical data

Ki-67 LI and PCNA LI were evaluated in a quantitive way using light microscopy. The Ki-67 LI and PCNA LI were defined as a percentage of positively stained tumor cells (brown reaction in the cell nuclei) among the total number of malignant cells assessed [29, 43]. Necrotic areas were excluded, as well as all inflammatory cells. Both Ki-67 LI and PCNA LI are presented as mean with standard deviation (SD). A cut-off >20% was used to define tumors with a high Ki-67 rate [44].

### Statistical analyses

The data was analyzed with GraphPad Prism 5.0 (USA) using an unpaired Student’s t-test and Mann Whitney U test to assess differences between grade II and III fibrosarcomas in PCNA and Ki-67 immunoreactivity. Spearman’s correlation test was used to correlate the histological grade of both sarcomas growth on the CAM and spontaneous feline fibrosarcomas with PCNA and Ki-67 LI. *P* < 0.05 were assigned as significant, while *p* < 0.01 and *p* < 0.001 were regarded as highly significant.

## Results

To characterize the growth kinetics of FFS1 and FFS3 cell line, the double proliferation time was calculated as 43 and 47 h for FFS1 and FFS3 cell line, respectively.

**Fig. 4:**
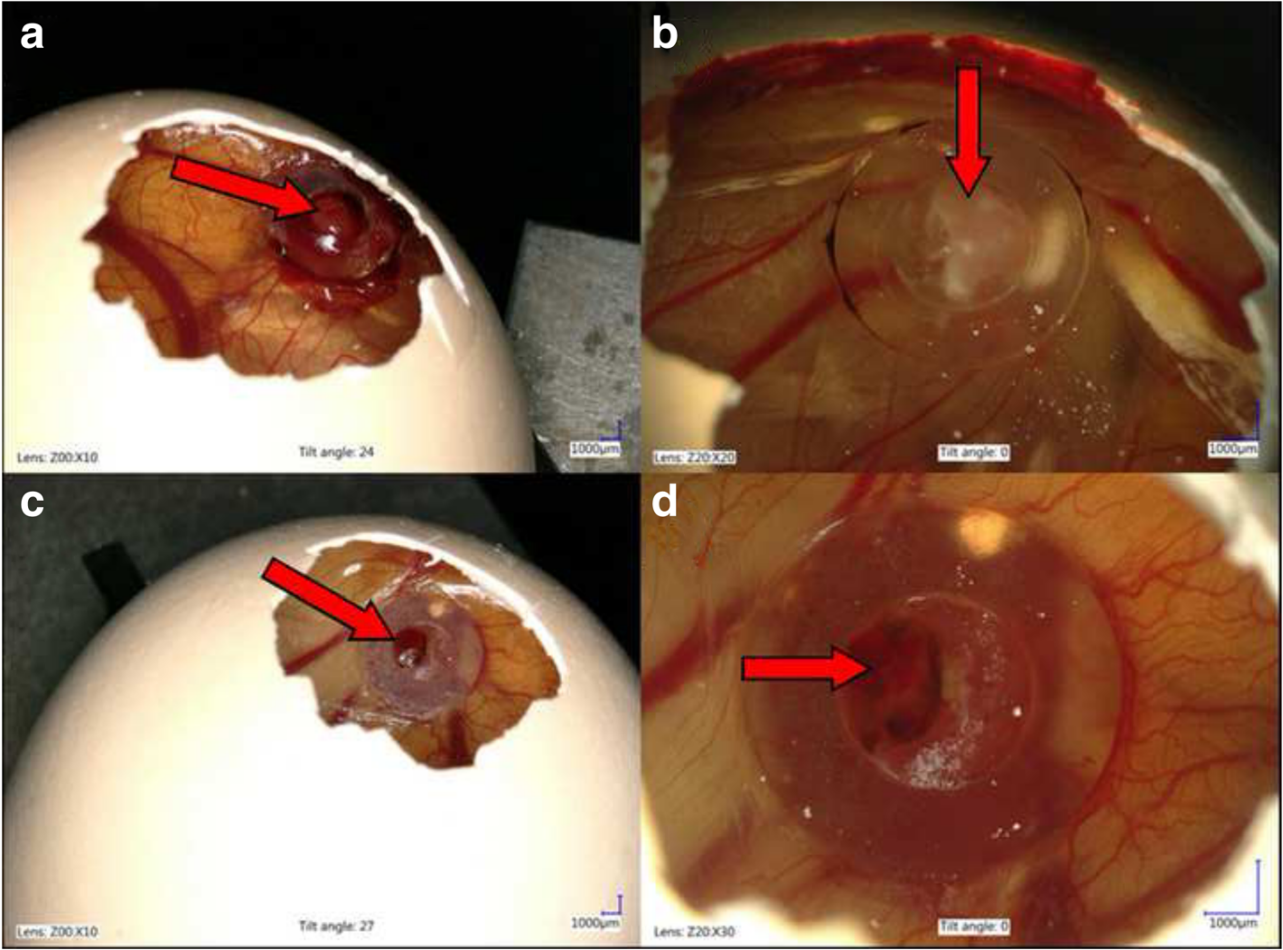
Feline fibrosarcomas from FFS1 (**a**, **b**) and FFS3 (**c**, **d**) cell line growth on the CAM

**Fig. 5:**
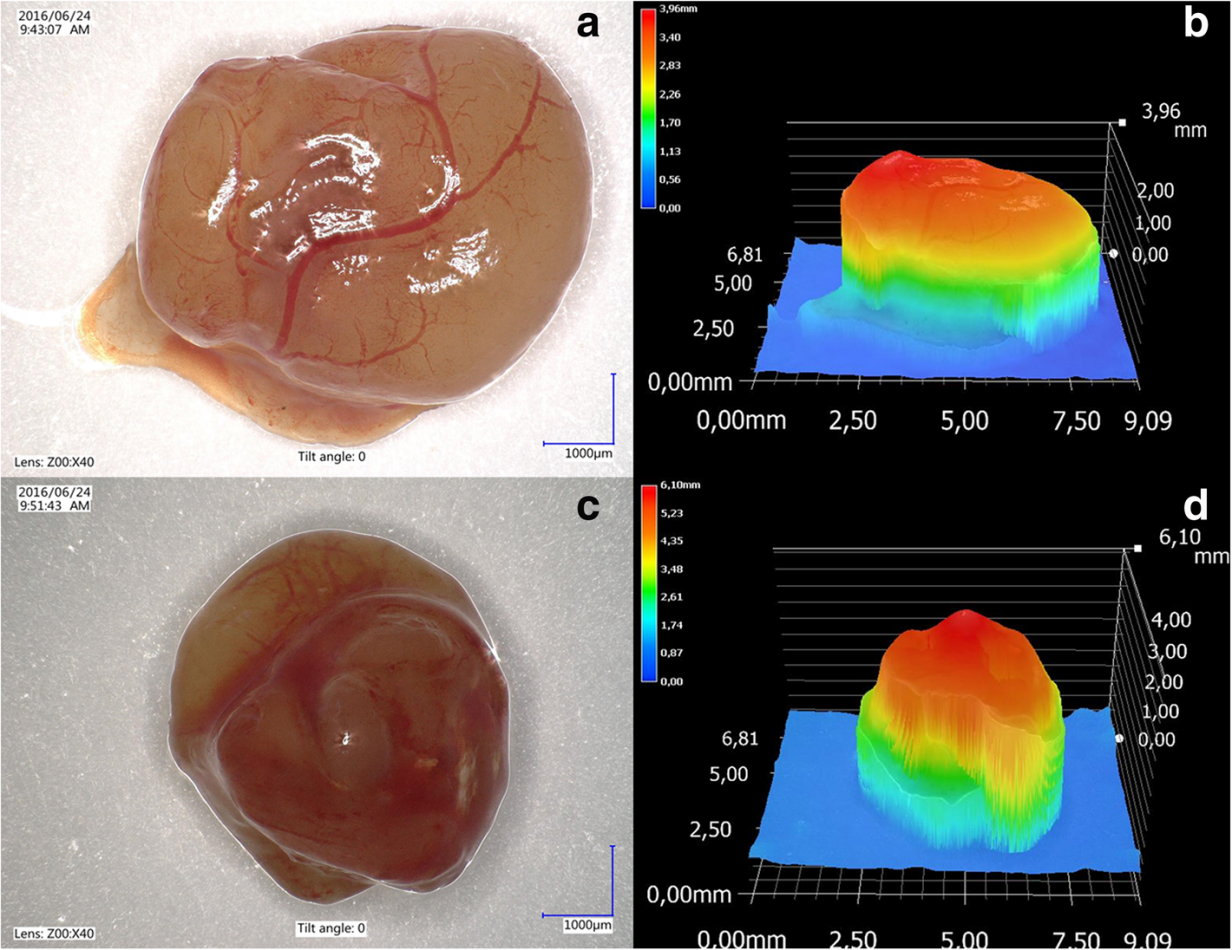
Isolated tumor from FFS1 cell line (**a**) and its 3D measurement (**b**) and isolated tumor from FFS3 cell line (**c**) and its 3D measurement (**d**)

Chick embryos mortality after neoplastic cells inoculation was 10% (3 of 30 embryos). Tumor growth was assessed in 23 of 27 chick embryos (78%). Tumor growth in or nearby silicon rings (Fig. 4). Blood vessels within tumors were always visible (Fig. 5). According to the Couto et al. grading system [40], the tumors from FFS1 and FFS3 cell lines were classified as grade III and grade II, respectively (Table 1). The shape of tumors from both cell lines was mainly oval and the surface was smooth (Fig. 5). Tumor consistency was elastic. Tumors from FFS1 cell line reached bigger size, which correlates with the results of the in vitro proliferation assay. Tumors from FFS1 cell line had on average 5.2 and 7.2 mm in diameter on the 16th and 19th day of incubation, respectively. Tumors from FF3 cell line had on average 3.6 and 5.2 mm in diameter in the 16th and 19th day of chick embryo incubation, respectively.

**Table 1:** Mitotic index, differentiation score, necrosis score and tumor grade for feline fibrosarcomas from FFS1 and FFS3 cell lines

	FFS1	FFS3
	n (%)	n (%)
Mitotic activity score		
1	0 (0)	13 (100)
2	0 (0)	0 (0)
3	10 (100)	0 (0)
Differentiation score		
1	0 (0)	0 (0)
2	0 (0)	0 (0)
3	10 (100)	13 (100)
Necrosis score		
1	3 (30)	6 (46.2)
2	7 (70)	7 (53.8)
3	0 (0)	0 (0)
Tumor grade		
I	0 (0)	0 (0)
II	0 (0)	13 (100)
III	10 (100)	0 (0)

*n* number of samples.

**Fig. 6:**
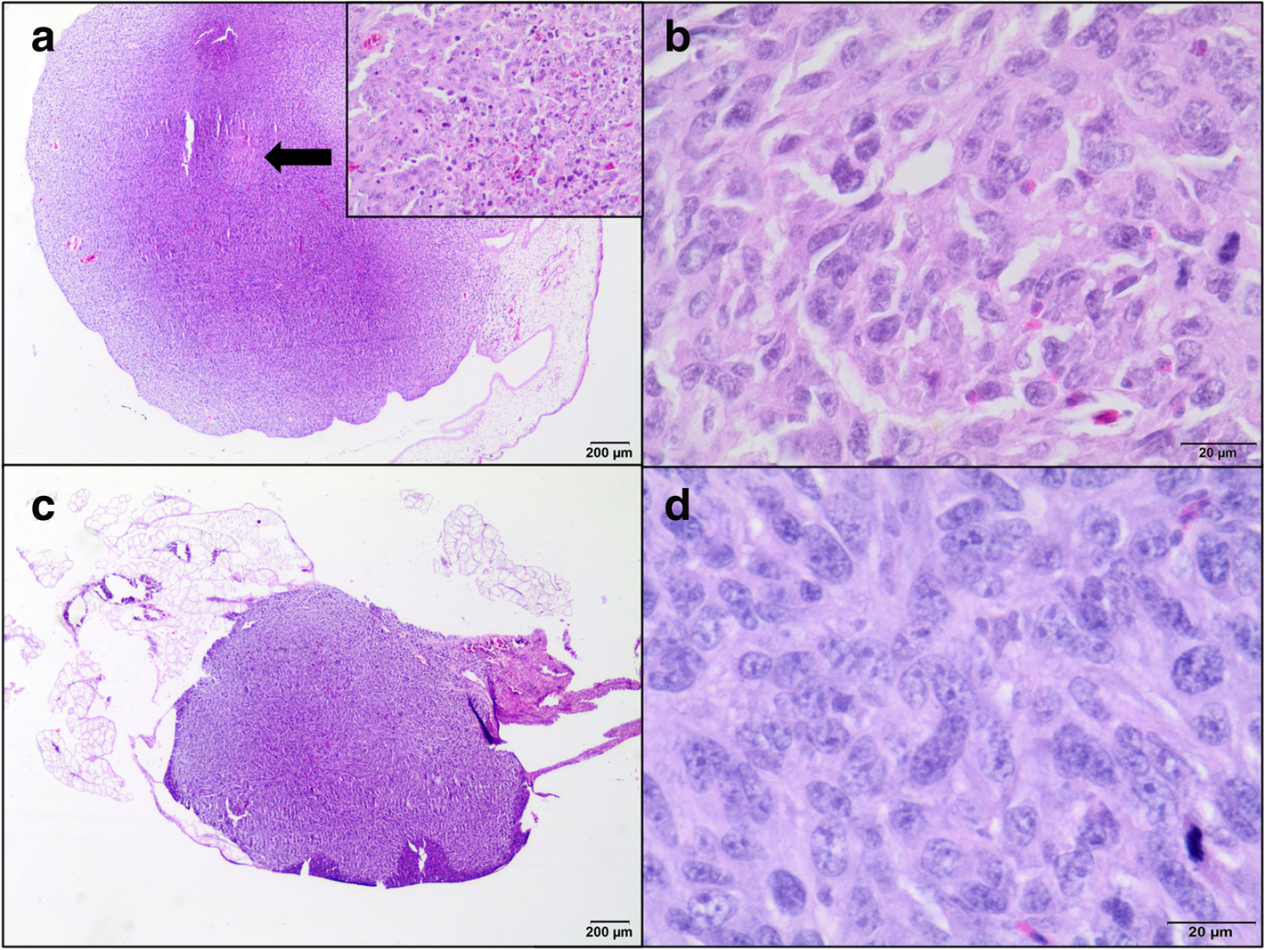
Hematoxillin and eosin staining of tumors growth from feline fibrosarcoma FFS1 and FFS3 cell lines on the CAM. **a** Unencapsulated, well-demarcated tumor mass of feline sarcoma FFS1 cell line on the CAM. Neoplastic cells demonstrate an invasive growth pattern and are able to penetrate the CAM. An area of necrosis is indicated by arrow. An insert image shows more details of necrosis: neoplastic cells undergo nuclear pycnosis and karyorrhexis. H&E, magnification 20× (200× insert)**. b** Higher magnification of tumor derived from FFS1 cell line. Densely cellular neoplasm composed of polygonal cells with indinstinct cell borders on a fine fibrovascular stroma. Neoplastic cells have a small to moderate amount of pale eosinophilic cytoplasm, round to elongate nucleus with variably distinct magenta nucleolus. Nuclear pleomorphism is marked. Mitotic figures average 2-4 per HPF with often bizarre mitoses. H&E, magnification 400×. **c** Tumor growth of feline sarcoma FFS3 cell line on the chick embryo chorioallantoic membrane, well-circumscribed, unencapsulated and invaded CAM. H&E, magnification 20×**. d** Higher magnification of tumor derived from FFS3 cell line. Dense pleomorphic cell population composed of neoplastic cells with indistinct cell borders supported by a fine fibrovascular stroma*.* Neoplastic cells have pale eosinophilic cytoplasm, an oval to elongate nucleus with finely stippled chromatin and a variably distinct magenta nucleolus (up to 2). The mitotic rate is up to 1-2 per HPF. H&E, magnification 400×

H&E staining confirmed the growth of 23 feline fibrosarcomas (10 and 13 tumors from FFS1 and FFS3 cell line, respectively) on the CAM (Fig. 6). Sarcomas derived from FFS1 and FFS3 were histologically similar, consisting of pleomorphic, oval or spindle-shaped cells forming interwoven bundles. Neoplastic cells had round to elongate nuclei with 1 up to 3 prominent nucleoli. They showed nuclear hyperchromasia. They had scant to abundant eosinophilic cytoplasm and indistinct cell borders*.* Some neoplastic cells were binucleated and had vacuolated cytoplasm. Tumors derived from the FFS1 cell line were characterized by higher mitotic rate (MI ranging from 19 to 25 per 10 HPFs, mean MI was 21.2) compared to tumors derived from the FFS3 cell line (MI ranging from 5 to 9 per 10 HPFs, mean MI was 7.9). All tumors contained area of necrosis (Tab. 1).

H&E staining of tumors derived from FFS1 and FFS3 cell lines confirmed that they were similar to spontaneous fibrosarcomas (high grade). They were composed of ovoid and spindle cells forming interwoven bundles, with the presence of collagenous stroma. Also areas of necrosis and mitotic figures were present. However, tumors derived from FFS1 and FFS3 cell lines were characterized by higher cellularity, had no multinucleated giant neoplastic cells and less collagen production than spontaneous fibrosarcomas.

**Fig. 7:**
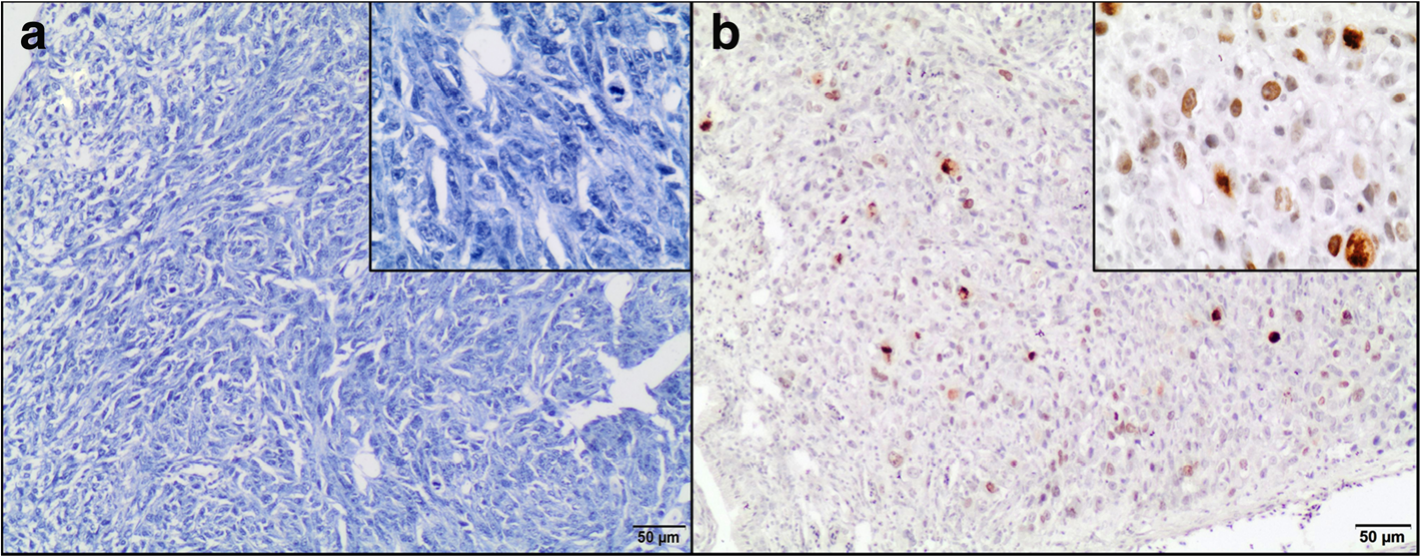
Ki-67 expression in feline fibrosarcomas from FFS1 (A) and FFS3 (B) cell line. **a** Negative Ki-67 expression. IHC, magnification 100× (400× insert). **b** Positive nuclear Ki-67 expression. Ki-67 revealed only faint immunoreactivity. IHC, magnification 100× (400× insert)

All tumors from the FFS1 cell line (grade III) had no immunoreactivity of Ki-67 (0%) (Fig. 7a), while tumors from the FFS3 cell line (grade II) showed none or low immunoreactivity of Ki-67 (Fig. 7b) (LI mean value was 5.2% (Table 2), LI ranges between 0 and 11.6%). There were no differences in Ki-67 immunoreactivity in tumors from FFS1 and FFS3 cell line as the Ki-67 rate for tumors from both cell lines was lower than 20%. Similarly, there was none or low immunoreactivity of Ki-67 in most (18 of 22 tumors) of spontaneous feline fibrosarcomas. Ki-67 LI mean value was 5.9% (Table 3) (LI ranges between 0 and 25.4%) and 9.8% (Table 3) (LI ranges between 0 and 26.4%) for grade II and III spontaneous fibrosarcomas, respectively.

**Table 2:** Correlation between tumor grade and proliferation markers (Ki-67, PCNA) in feline fibrosarcomas from FFS1 and FFS3 cell lines

Labeling index	Tumor grade		*p* value	r value
	Grade III (tumors from FFS1 cell line) (*n* = 10)	Grade II (tumors from FFS3 cell line) (*n* = 13)		
Mean Ki-67 (SD)	0% (0)	5.% (3.1)	-	-
Mean PCNA (SD)	77.9% (8.9)	53.7% (8.0)	<0.001	0.8247

*SD* standard deviation, *n* number of samples

**Table 3:** Correlation between tumor grade and proliferation markers (Ki-67, PCNA) in spontaneous feline fibrosarcomas

Labeling index	Tumor grade		*p* value	r value
	Grade III (*n* = 9)	Grade II (*n* = 14)		
Mean Ki-67(SD)	9.8% (10.6)	5.9% (9.6)	0.2594	0.2452
Mean PCNA (SD)	86.6% (10.3)	75.2% (12.6)	0.0194	0.4837

*SD* standard deviation, *n* number of samples

**Fig. 8:**
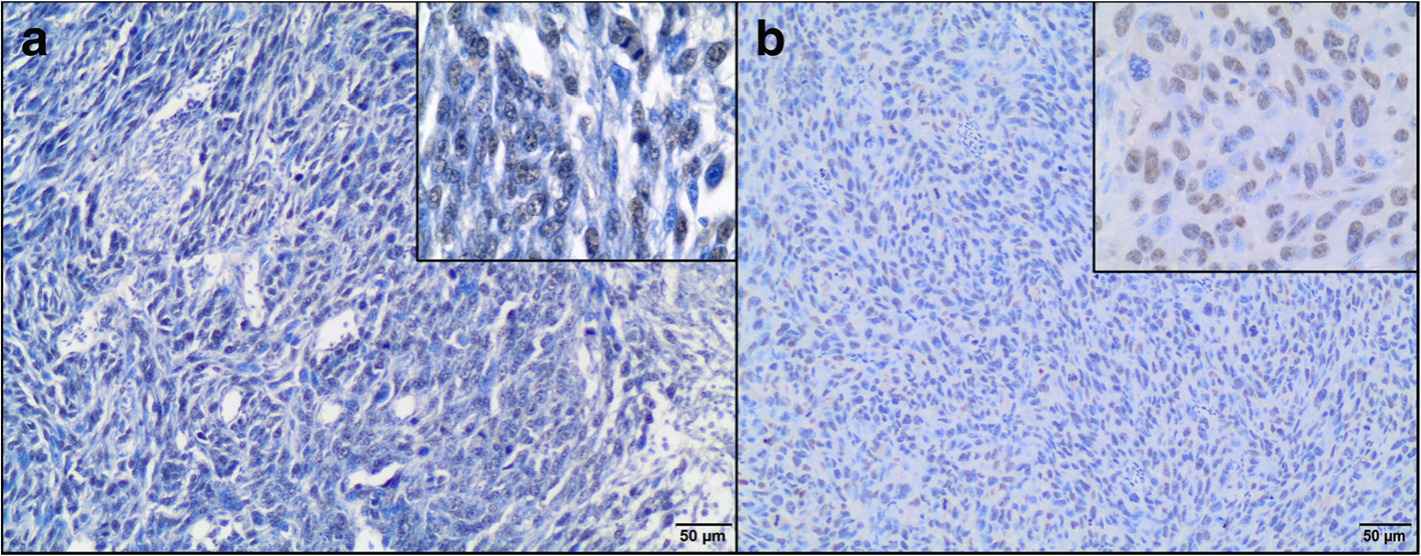
PCNA expression in feline fibrosarcomas from FFS1 (**a**) and FFS3 (**b**) cell line. **a** and **b** Positive nuclear PCNA expression*.* IHC, magnification 100× (400× insert)

**Fig. 9:**
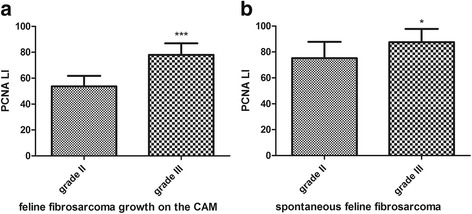
Statistical analyses of PCNA immunoexpression in tumors grown from FFS1 (grade III) and FFS3 (grade II) cell line on the CAM (**a**) and in spontaneous feline fibrosarcomas (grade III and II) (**b**). *P* < 0.05 was assigned as significant (*), while *p* < 0.001 was assigned as highly significant (***)

Higher PCNA expression (LI mean value was 77.9% (Table 2), LI ranges from 70 to 93.7%) was in tumors from FFS1 cell line (grade III) (Fig. 8a), while in tumors from FFS3 cell line (grade II) the mean value of PCNA expression was 53.7% (Table 2) (ranges from 37.9 to 62.6%) (Fig. 8b). There was a statistically important difference in PCNA immunoreactivity between tumors grown from FFS1 and FFS3 cell line (*p* < 0.001) (Fig. 9a). Moreover, the higher PCNA LI was positively correlated with the higher tumor grade (*r* = 0.8247; *p* < 0.001). In spontaneous feline fibrosarcomas PCNA LI mean values were: 86.6 and 75.2% for grade III and grade II, respectively (Table 3). There was a statistically significant difference (*p* < 0.05) in PCNA immunoreactivity between grade II and grade III spontaneous feline fibrosarcomas (Fig. 9b). In spontaneous feline fibrosarcomas the higher PCNA LI also positively correlates with the higher tumor grade (*r* = 0.4837, *p* < 0.05).

## Discussion

In vitro studies are needed for screening drug efficiency first, however, many drugs that have positive results in vitro fail during clinical trials [45]. It is obvious that preclinical in vivo studies are essential for discovering new therapeutic agents, however, rodent models are time consuming, expensive and require approval from the Animal Ethical Commission. Recently, the CAM model has been utilized for various tumors in human medicine. Our study demonstrates that both examined fibrosarcoma cell lines (FFS1 and FFS3) can form solid, vascularised tumors on the CAM. It is in agreement with our previous study describing tumors growth from FFS1WAW cell line [20].

The histopathology of tumors grown on the CAM was similar to spontaneous feline fibrosarcomas. Similarly to spontaneous fibrosarcomas (high*-*grade) tumors derived from FFS1 and FFS3 cell lines had some areas of necrosis and many mitotic figures. Tumors grown on the CAM had higher cellularity, less collagen production and no multinucleated giant neoplastic cells in comparison to spontaneous feline sarcomas. Collagen production was less pronounced on the CAM, likely due to a shorter time for tumor growth (only 13 days) than in spontaneous sarcomas. In case of spontaneous sarcomas we know from the history of the patients that tumors were detected by the owners at least 3 months before surgical resection (data not shown). Giant neoplastic cells were not found, however, according to the literature they are found in 50% of FISS and only in 11% of spontaneous feline sarcomas [40, 46].

We further improved the methodology by chick embryo turn-over on the 3rd day of incubation, which decreased the embryos mortality from 30% [20] to 10% as no manipulations with the parchment-like membrane were needed. Tumors were classified as present when their diameter was at least 2 mm (according to classification suggested by Balke et al.) [16]. Tumors from both cell lines were visible on the 16th day of chick embryo incubation, which is in agreement with our previous study [20], where we stated that FFS1WAW fibrosarcoma cell line needs 10 days to form solid tumors on the CAM. This model may be used not only as a preclinical model to better understand the biology of tumors, but also to study the effectiveness of new drugs, as an intermediate model between in vitro studies and in vivo studies, what we have reported in our latest article on the use of glutathione-stabilized gold nanoparticles conjugated to doxorubicin (Au-GSH-Dox) [21].

There are three main critical points in such experiments: making a “window” in the eggshell, inoculating cancer cells and measuring tumor growth. Mechanic manipulations should be done with extreme caution as inappropriately done may lead to death of the chick embryos. Inoculating neoplastic cells should be done directly after trypsinization of cells (cells should not reach full confluence) and should last no longer than 30 min as it may result in lower percentage of tumor growth. In the last day of the experiment, the tumor size obtained by the computer software should be correlated with the tumor size obtained by the caliper, after removing tumors from the eggs. If tumor size obtain with the caliper does not correlate with the size obtained by the computer software, those tumors should be excluded from the study. The use of Peira Tumor Scanner, positron emission tomography (PET) or computer tomography (CT) may be also consider, however, it enlarges the cost of such study.

The main advantage of the CAM model for preclinical oncological studies in comparison to rodent model is the ethical point as presented model follows “3R” rules and enables to reduce amount of animals used for further in vivo studies. Moreover, in many countries, there is no need to apply for Animal Ethics Commission Approval. The other advantages are: relatively simple experimental approach, fast tumor growth and cost-efficiency. However, the disadvantages as unfeasible long-term tumor observation and lack of competent inflammation should be considered [21]. Despite drug testing, the CAM model has been utilized for pro- and antiangiogenic studies [10, 12, 18, 47] and for acute toxicological studies on anticancer drugs [48]. Both *in ovo* and e*x ovo* (shell-less) model is used to assess metastatic potential of various cancer cell lines [18, 49].

Recently veterinary studies have focused on cellular proliferation in tumor as a useful factor for understanding the biology of tumors and as a prognostic marker for overall survival and for the results of chemotherapy treatment. However, to the best knowledge of the authors, there are only a few studies on the use of Ki-67 as a proliferation marker in FISS [38, 50]. As a result, we described the proliferation patterns for these tumors using the 3D CAM model. In our study tumors from FFS1 cell line (grade III) had no expression of Ki-67, while the fibrosarcomas from FFS3 cell line (grade II) showed the highest expression of Ki-67 for 11,6% and no correlation between Ki-67 index and tumor grade was noted. Also most of spontaneous feline fibrosarcomas (grade II and III) had no or low (Ki-67 LI was <20% in 19 of 23 tumors) expression of Ki-67. The average Ki-67 LI in all tested spontaneous fibrosarcomas was 7.3%. It is in agreement with the study performed by Eckstein et al., who also demonstrated no correlation between Ki-67 expression and tumors grade for FISS. In their studies, Ki-67 expression was between 10 and 40% (on average 14%) [39]. In the study presented by Sysel et al. the average Ki-67 LI for vaccine-associated fibrosarcomas was 32% [50], which is higher than in our study, but only three vaccine associated-sarcomas were included and the authors did not examine the histological grades of tumors.

Also in studies performed by Nowak et al. on 27 canine fibrosarcomas, 70% of tumors have no expression of Ki-67, 22% of tumors have low Ki-67 expression, and only 4% of tumors have both intermediate and high Ki-67 expression. They showed a weak correlation between expressions of Ki-67 and methallothonein (MT), which was shown to correlate with tumor grading in various tumors in human medicine [51]. Although in human studies on soft tissue sarcomas a good correlation between Ki-67 and tumor grade was shown [52, 53], the pathogenesis and etiology of FISS is entirely different than for human soft tissue sarcoma. In our opinion a reliable comparison should only be made with FISS results, although many scientists compare FISS with soft tissue sarcomas of various species, probably due to a lack of enough studies on FISS.

In our study PCNA LI was much higher for fibrosarcomas from both cell lines than Ki-67 LI. This is in agreement with most of the studies on proliferation markers in various tumors e.g. canine lung carcinomas [34]. This may be because PCNA can be demonstrated first in the middle G1 phase of the cell cycle, reaching a maximum in S phase and declining in G2 phase. This protein is not expressed during mitosis, but it is detectable throughout the proliferating cell cycle due to its long half-life (about 90 h) [34]. Even if PCNA is considered a relatively S phase specific antigen, it is present for longer periods than Ki-67 in the nucleus during the cell cycle. PCNA is also expressed in non-cycling cells in association with DNA repair [32]. Low expression of Ki-67 LI in our study may be interpreted by the fact that the cells were in the beginning of the G1 phase which cannot be stained with anti-Ki-67 antibody.

Interestingly, we demonstrated that the PCNA index revealed a high positive correlation (*r* = 0.8247, *p* < 0.001) with histologic grading of sarcomas grown on the CAM, as opposed to Ki-67 where the expression was <20% in both groups of tumors. The positive correlation (*r* = 0.4837, *p* < 0.05) with histological grading was also observed for spontaneous feline fibrosarcomas. In most studies, both in human and veterinary medicine, Ki-67 seems to be a more specific proliferation marker than PCNA, however, the immunoreactivity of Ki-67 and PCNA varies in different types of tumors [54–56]. Unfortunately, there is lack of the studies on the PCNA immunoreactivity in FISS. Our study indicates that PCNA overexpression may be associated with histological grade of FISS, although further studies are needed to confirm this hypothesis.

There are a few limitations of our study which should be taken into consideration. First of all, tumors from only two cell lines (FFS1, FFS3) were examined and classified as grade II and III fibrosarcomas. We did not have grade I fibrosarcomas, which remains unable to correlate with Ki-67 and PCNA immunostaining. Moreover, only 23 feline spontaneous fibrosarcomas were used as positive control. In order to clearly evaluate the role of proliferation markers for clinical outcome, further studies with xenograft tumor model (CAM model), spontaneous tumors and preferably monitoring animals from which those tumors were taken are needed. However, the CAM model owing to its low cost, relative ease to perform and no approval requirement from the Animal Ethics Commission seems to be a good alternative to the rodent model to extend the knowledge on fibrosarcoma biology and may be successfully used for further histopathological and molecular studies.

## Conclusions

The authors demonstrated that two feline fibrosarcoma cell lines (FFS1 and FFS3) can form solid tumors on the CAM and that the CAM model can be successfully used for histopathological analyses in order to expand the knowledge on tumor biology. We have demonstrated that feline fibrosarcomas (both grade II and III) have none or very low expression of Ki-67. On the other hand, tumor grade positively correlates with PCNA expression, what indicates that PCNA may be a better marker to assume tumor grade in feline fibrosarcomas, in opposed to Ki-67. However, further investigation of proliferation marker, in bigger number of feline spontaneous fibrosarcomas and feline fibrosarcomas grown on the CAM from different cell lines, is needed to confirm these observations.

## Abbreviations

Au-GSH-Dox: Glutathione-stabilized gold nanoparticles conjugated to doxorubicin
CAM: Chorioallantoic membrane
CT: Computer tomography
DMEM: Dulbeco’s Modified Eagle Medium
FBS: Fetal bovine serum
FeLV: Feline leukemia virus
FISS: Feline injection-site sarcoma
H&E: Hematoxylin and eosin
IHC: Immunohistochemistry
LI: Labeling index
MI: Mitotic index
MT: Methallothonein
PCNA: Proliferating nuclear cell antigen
PET: Positron emission tomography
SD: Standard deviation
